# A disparity in the number of studies related to COVID-19 and SARS-CoV-2 between low- and middle-income countries and high-income countries

**DOI:** 10.1093/inthealth/ihaa088

**Published:** 2020-10-31

**Authors:** Takuma Usuzaki, Shuji Chiba, Minoru Shimoyama, Mami Ishikuro, Taku Obara

**Affiliations:** Tohoku University School of Medicine 2-1 Seiryo-machi, Aoba-ku, Sendai, Miyagi 980-8573, Japan; Dokkyo Medical University 880 Kitakobayashi, Mibu, Shimotsugagun, Tochigi 321-0293, Japan; Tohoku University School of Medicine 2-1 Seiryo-machi, Aoba-ku, Sendai, Miyagi 980-8573, Japan; Department of Preventive Medicine and Epidemiology, Tohoku Medical Megabank Organization, Tohoku University 2-1, Seiryo-machi, Aoba-ku, Sendai, Miyagi 980-8573, Japan; Department of Molecular Epidemiology, Graduate School of Medicine, Tohoku University 2-1, Seiryo-machi, Aoba-ku, Sendai, Miyagi 980-8573, Japan; Department of Preventive Medicine and Epidemiology, Tohoku Medical Megabank Organization, Tohoku University 2-1, Seiryo-machi, Aoba-ku, Sendai, Miyagi 980-8573, Japan; Department of Molecular Epidemiology, Graduate School of Medicine, Tohoku University 2-1, Seiryo-machi, Aoba-ku, Sendai, Miyagi 980-8573, Japan; Department of Pharmaceutical Sciences, Tohoku University Hospital 1-1 Seiryo-machi, Aoba-ku, Sendai, Miyagi 980-8574, Japan

## Abstract

**Background:**

There may be a difference in the number of articles about COVID-19 and SARS-CoV-2 between low- and middle-income countries (LMICs) and high-income countries (HICs).

**Methods:**

We analyzed authors’ affiliations from 36 432 articles related to COVID-19 and SARS-CoV-2. We introduced logarithmic density and compared the number of articles and logarithmic density of LMICs with those of HICs.

**Results:**

The number of articles and the logarithmic density of LMICs were lower than those of HICs (p<0.0001 for both).

**Conclusions:**

There was a disparity in the number of articles related to COVID-19 and SARS-CoV-2 between LMICs and HICs.

## Introduction

The coronavirus disease 2019 (COVID-19), caused by severe acute respiratory syndrome coronavirus 2 (SARS-CoV-2), has spread to almost all the countries of the world and efforts have been made to reduce transmission. Previous studies^1^^,^^2^ analyzing the current COVID-19 pandemic situation in low- and middle-income countries (LMICs) have pointed out that support from the international community to LMICs is needed in relation to the COVID-19 outbreak. One of the reasons why LMICs are in need of support is that a shortage of human, material and financial resources may prevent studies from being conducted in LMICs and evidence being accumulated^3^; that is, there may be a difference in the number of articles about COVID-19 and SARS-CoV-2 between LMICs and high-income countries (HICs).^2^

## Materials and methods

To evaluate if there is a difference in the number of articles about COVID-19 and SARS-CoV-2 between LMICs and HICs, we analyzed data from the COVID-19 Open Research Dataset Challenge (https://www.kaggle.com/allen-institute-for-ai/CORD-19-research-challenge), which collected 233 284 articles of all types related to COVID-19, SARS-CoV-2 and coronavirus. From these articles, we analyzed those published from 31 December 2019, when the first cluster of cases was reported in Wuhan,^4^ and 28 August 2020, by country and region. We used authors’ affiliations to determine the country in which a study was performed. When an article was written by more than one author from the same country, it was counted as a single country. The World Bank country classification was used to categorize each country as either an LMIC or an HIC. We used the Novel Corona Virus 2019 Dataset (https://www.kaggle.com/sudalairajkumar/novel-corona-virus-2019-dataset) to obtain the total number of infected people in each country as of 28 August 2020. To adjust the number of articles by the number of infected people, we introduced a logarithmic density value, which is the number of articles divided by the common logarithm of the number of infected people. Logarithmic density is defined to ensure linearity for an exponential increase in infected people and can be interpreted as an analog of the number of studies per infected person. The number of articles in LMICs was compared with that in HICs by Mann–Whitney U test. The Pearson correlation coefficient between the number of articles and logarithm of the number of infected people was analyzed for both LMICs and HICs. We calculated a logarithmic density value for each country where the number of infected people was greater than one. We compared the mean of logarithmic density for LMICs with those for HICs by Mann–Whitney U test.

## Results

**Figure 1.**
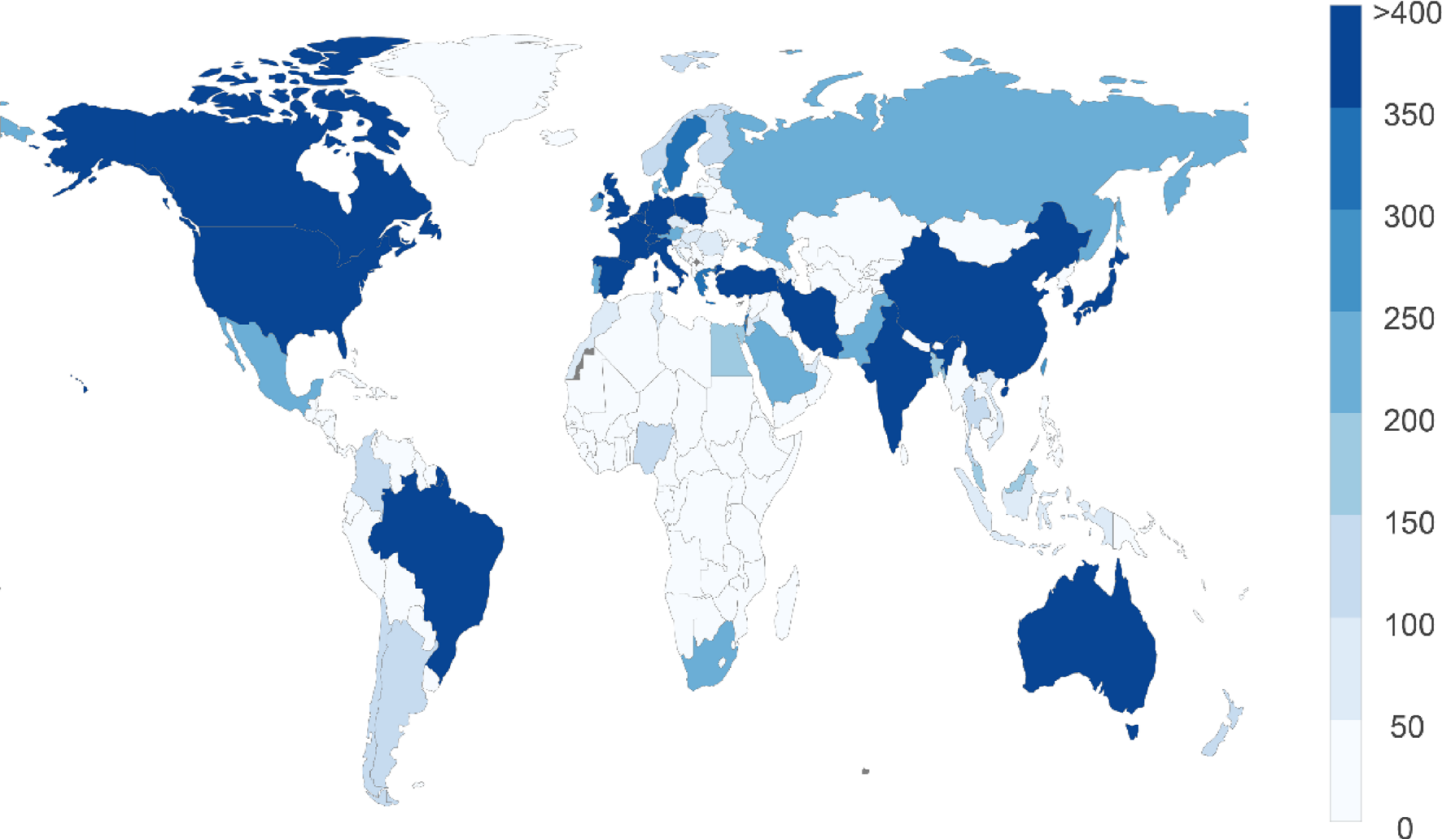
A world choropleth map of the number of articles related to COVID-19 and SARS-CoV-2 published from 31 December 2019 to 28 August 2020 by country. The number of countries that published 0–50, 51–100, 101–150, 151–200, 201–250, 251–300, 301–350, 351–400 and >400 articles was 124, 13, 8, 4, 8, 1, 4, 1 and 18, respectively.

We found that 36 432 articles were published from 96 (69.1%) LMICs and 52 (98.1%) HICs. The number of articles published from LMICs and HICs was 11 304 and 25 128, respectively. The number of articles published from LMICs was lower than that of HICs (p<0.0001). Figure 1 shows a world choropleth map of the number of articles related to COVID-19 and SARS-CoV-2 published from each country during the study period. The number of articles was associated with the logarithm of the number of infected people (Pearson correlation coefficient r=0.25, p=0.0041 and r=0.59, p<0.0001 for LMICs and HICs, respectively). The logarithmic density was calculated for 128 LMICs and 53 HICs. The median of logarithmic density in LMICs and HICs was 1.01 and 32.0, respectively. The mean logarithmic density of LMICs was lower than that of HICs (p<0.0001).

## Discussion

In our analyses, the number of articles related to COVID-19 and SARS-CoV-2, and the mean logarithmic density in LMICs, were both lower than those of HICs. Research is key to understanding the characteristics of COVID-19, including its transmission dynamics, spectrum of illness and outcomes, the impact of comorbidities and common coinfections.^2^ Surveillance and rapid cycle research on the effects of pandemic response strategies can lead to reductions in transmission. Any human, material or financial support from the international community^1^ could assist with conducting studies in LIMCs and thus contribute to overcoming this global pandemic. In addition to resource shortages in LMICs, individual know-how and organizational practices in publishing may be insufficient compared with HICs. Previous research highlighted that the pattern of the spread of SARS-CoV-2 was different in Africa because of epidemiological factors.^5^ Further studies should be conducted to evaluate the difference and help deal with the pandemic.

Our study has some limitations. First, both the number of articles and logarithmic density might not directly reflect the control status of the pandemic in a country. LIMCs may have insufficient reporting systems and the number of infected people may be underestimated. Second, we included all types of articles in the analysis such as editorials, opinion pieces and commentaries, which do not constitute original research. However, our results revealed that LIMCs have a lower capacity with which to disseminate internal situations concerning COVID-19 than HICs and quantitatively confirmed that LIMCs may be in need of support from the international community to conduct studies related to COVID-19 and SARS-CoV-2, as has been previously argued.^1^^,^^2^

## Data Availability

The data that support the findings of this study are openly available in COVID-19 Open Research Dataset Challenge (CORD-19) and Novel Corona Virus 2019 Dataset at https://www.kaggle.com/allen-institute-for-ai/CORD-19-research-challenge and https://www.kaggle.com/sudalairajkumar/novel-corona-virus-2019-dataset, respectively.
